# Growth estimates of Caribbean reef sponges on a shipwreck using 3D photogrammetry

**DOI:** 10.1038/s41598-019-54681-2

**Published:** 2019-12-05

**Authors:** Lauren K. Olinger, Alexander R. Scott, Steven E. McMurray, Joseph R. Pawlik

**Affiliations:** 1 Department of Biology and Marine Biology, University of North Carolina Wilmington, Wilmington, North Carolina USA; 2 Department of Wildlife, Fish, and Conservation Biology, University of California Davis, Davis, California USA

**Keywords:** Population dynamics, Marine biology

## Abstract

The growth rates and ages of many benthic marine organisms are poorly understood, complicating our understanding of ecosystem change. This is particularly true for sponges, which are morphologically diverse and lack indicators of annual growth. In this study, we used emerging technologies to measure volume, surface area, and approximate age of 16 sponge species on the Tibbetts shipwreck off Cayman Brac, Caribbean Sea. Photogrammetry was used to determine the volume of individual sponges on the wreck surface, and a time series of YouTube videos was amassed in order to approximate the greatest possible age of the sponges as 8.74 y. Applying the volume measurements to an existing growth equation for the Caribbean sponge *Aiolochroia crassa* yielded age estimates of 5.2–10.4 y for the largest individuals of the 16 species. Specific growth rates were then calculated for 7 species from the Tibbetts and 8 species from a second shipwreck (Spiegel Grove, Key Largo, FL). Subsequent growth forecasts from these 15 species corroborate a resource trade-off between growth and the production of chemical defenses. Shipwrecks and other anthropogenic structures can be an important source of demographic information for benthic organisms, provided that certain assumptions about their provenance and history can be met.

## Introduction

A better understanding of marine ecosystem function and change requires knowledge of the demographics of the organisms that make up marine communities. Unfortunately, many benthic marine organisms lack clear indicators of annual growth that can be used to determine their growth rates and ages, including many modular and clonal species such as sponges, corals, and ascidians. Additionally, and at a more basic level, measurements of the abundance of benthic marine organisms are most commonly determined as two-dimensional (2D) percentage cover of the substratum, despite the fact that many organisms have complex, three-dimensional (3D) morphologies (e.g., branching corals)^1^ and substantial biomass that is poorly represented by 2D metrics (e.g., massive emergent sponges)^2^. Past approaches to measuring volume and surface area have included displacing subject organisms in water^3,4^ and wrapping organisms with paraffin wax or tinfoil^5,6^. However, these traditional methods require destructive (and often fatal) removal of the organism from the substratum, therefore limiting replicates and precluding repetitive time-series measurements. Less destructive methods for measurements of volume and surface area include planar projection photography^7^ or the calculation of volume from component geometric shapes determined from linear measurements of the dimensions of the organism^8,9^. These methods can be difficult and time-consuming, and are best applied to organisms with simple morphologies and large sizes, for which measurement error is likely to have a small effect on volume determination^10^.

An emerging technology for the measurement of surface area and volume of benthic organisms is 3D photogrammetry. Earlier 3D imaging techniques such as stereo-photogrammetry required bulky dual-camera equipment and technical expertise, but it is now possible to produce high-resolution digital models with a single camera and little specialized training^11,12^. This ‘‘structure from motion’’ photogrammetry is a result of increasing computational power and advancements in imaging and algorithm processing. Furthermore, high-quality, reasonably-priced, and compact waterproof cameras such as GoPros are now widely available to recreational scuba divers and scientific researchers^12^. Continued improvements in photogrammetric processing software and lower cost cameras have led to the increased application of photogrammetry in the aquatic sciences.

For studies of coral reefs, photogrammetry has primarily been used to produce models of reef-building corals and to measure reef-scale structural complexity^13,14^, with only limited application to other organisms^15^. However, this technology is particularly well suited to measuring the volume (a proxy for biomass) of sponges on coral reefs. Unlike many reef organisms that consist of thin blades or sheets of living tissue (macroalgae, reef-building corals) for which 2D measurements are good proxies for relative abundance, sponges are morphologically complex and often have robust bodies with thick tissue. Therefore, while estimates of benthic percentage cover on reefs across the Caribbean provide similar values for corals and sponges (about 16%)^16^, the difference in biomass for sponges is likely orders of magnitude larger^2,10^. With reports of increasing abundances of sponges on Caribbean reefs^17–20^, and growing interest in the effects of sponges on ecosystem processes that include carbon and nutrient cycling^21–24^, there is a clear need to gather better basic demographic information on common Caribbean reef sponge species. Additionally, from the standpoint of natural resource management and conservation, sponges are often excluded from environmental impact assessments (EIAs) and concomitant economic valuation and mitigation for loss, largely due to a lack of basic demographic data for most sponge species^25^.

One approach to determining the age of modular or clonal benthic organisms is to measure their volume on artificial reef structures, where the greatest approximate age of the organisms colonizing them can be known; good examples are shipwrecks with known dates of sinking^4,26^. In this study, we generated 3D models using photogrammetry to measure the volume and surface area of individual sponges growing on the Tibbetts shipwreck, located off the west coast of Cayman Brac, in the Caribbean Sea. Publicly-available footage of this shipwreck, posted on YouTube by recreational divers and professional photographers, allowed us to define the earliest date of sponge recruitment, from which we approximated the age of the largest individuals. For each species, we compared this approximate age to one calculated using an existing exponential equation describing the growth of the sponge *Aiolochroia crassa*^27^. Lastly, we calculated and compared specific growth rates for the most abundant sponge species from the Tibbetts wreck to those from the Spiegel Grove wreck (Key Largo, FL), whose displacement volumes were previously measured^4^.

## Results

**Figure 1:**
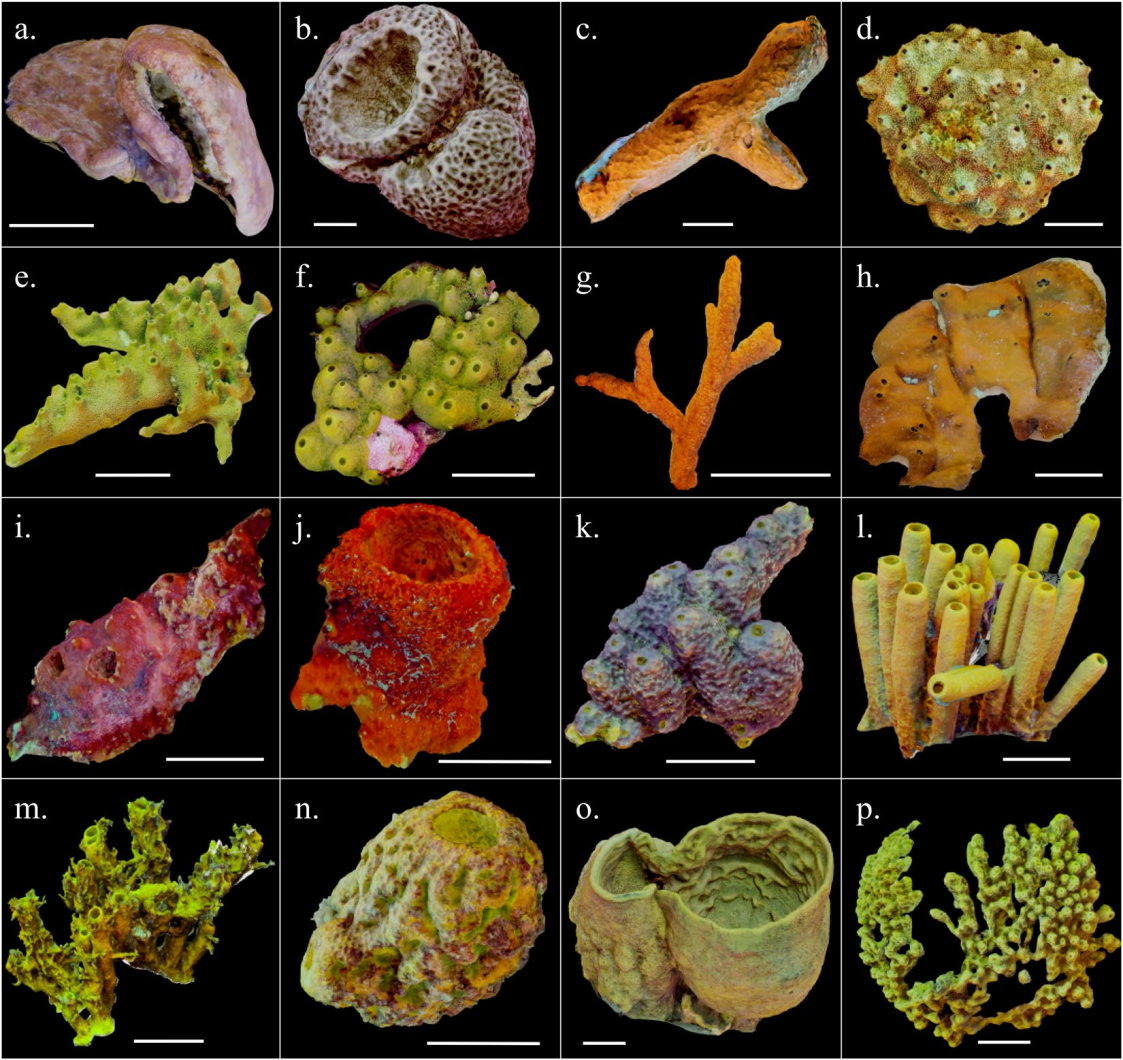
Screenshots of 3D models (textured meshes) of a representative individual from each sponge species from the Tibbetts shipwreck. White bar denotes 10 cm scale. (**a**). *Agelas dilitata*; (**b**). *Geodia neptuni*; (**c**). *Chondrosia reniformis*; (**d**). *Ircinia felix*; (**e**). *Smenospongia aurea*; (**f**). *Smenospongia conulosa*; (**g**). *Ptilocaulis walpersii*; (**h**). *Plakortis sp*.; (**i**). *Neofibularia nolitangere*; (**j**). *Mycale laxissima*; (**k**). *Aiolochroia crassa*; (**l**). *Aplysina fistularis*; (**m**). *Aplysina insularis*; (**n**). *Aplysina lacunosa*; (**o**). *Verongula gigantea*; (**p**). *Verongula rigida*.

**Figure 2:**
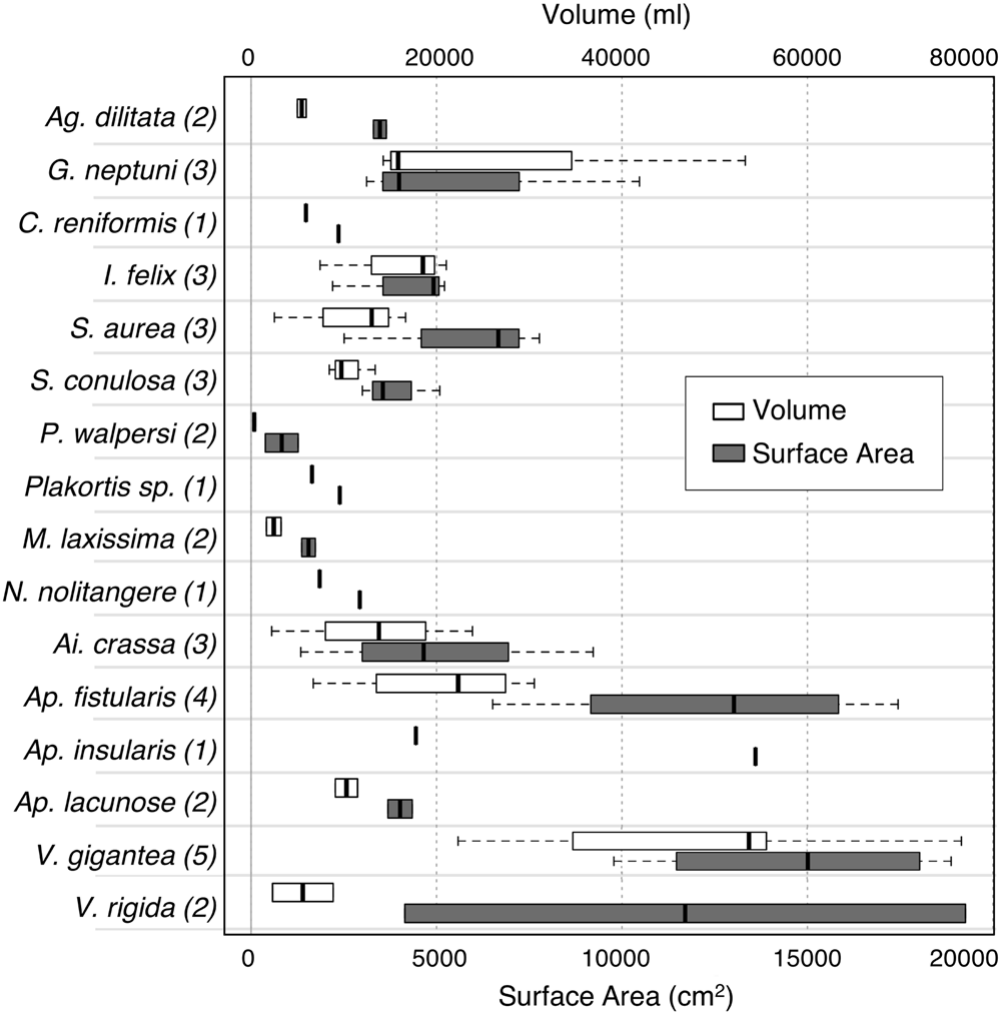
Volume and surface area data for each sponge species from the Tibbetts shipwreck. The number of replicates for each species is shown in parentheses. Thick vertical line denotes median, and maximum and minimum values are indicated by ends of whiskers (n > 2), ends of boxes (n = 2), or thick vertical lines (n = 1).

We produced 41 models in total; these represented 38 individual sponges belonging to 16 species plus three replicates of one of these individuals (*Verongula gigantea*) used for precision analysis (Fig. 1). Size measurements were highly variable across all 38 individuals, with 392-fold variation in volume and 50-fold variation in surface area. The three largest individuals by volume (V) belonged to the massive vase species *Verongula gigantea* (V = 76,641, 55,630, 53,736 ml), and the three smallest individuals belonged to the branching species *Ptilocaulis walpersi* (V = 196, 479 ml) and vase-forming species *Mycale laxissima* (V = 1,645 ml). The three individuals with the greatest surface area (SA) belonged to the massive lobate species *Verongula rigida* (SA = 19,262 cm${}^{2}$) and *V. gigantea* (SA = 18,884, 18,137 cm${}^{2}$), and the three individuals with the smallest surface area belonged to *P. walpersi* (SA = 383, 1,270 cm${}^{2}$) and *Aiolochroia crassa* (SA = 1,340 cm${}^{2}$; Table 1, Fig. 2).

**Table 1:** Volumes and surface areas of each sponge from the Tibbetts wreck and estimated ages for the largest individuals from each species based on the exponential growth model of Wilkinson and Cheshire (1988). Chemically defended species in bold

Order	Species	#	V (ml)	SA (cm${}^{2}$)	Est. age (y)
Agelasida	***Agelas dilitata***	1	5,954	3,647	7.8
		2	4,984	3,298	—
Astrophorida	*Geodia neptuni*	1	53,296	10,470	10
		2	15,863	3,990	—
		3	14,257	3,115	—
Chondrosida	*Chondrosia reniformis*	1	5,919	2,357	7.8
Dictyoceratida	***Ircinia felix***	1	21,047	4,918	9.1
		2	18,525	5,214	—
		3	7,436	2,197	—
	***Smenospongia aurea***	1	16,663	6,667	8.8
		2	13,001	7,774	—
		3	2,516	2,508	—
	***Smenospongia conulosa***	1	13,387	5,086	8.6
		2	9,729	3,000	—
		3	8,436	3,553	—
Halichondrida	***Ptilocaulis walpersi***	1	479	1,270	5.2
		2	196	383	—
Homosclerophorida	***Plakortis sp****.*	1	6,569	2,391	7.9
Poecilosclerida	***Neofibularia nolitangere***	1	3,245	1,732	7.2
	***Mycale laxissima***	1	1,645	1,364	8
		2	7,390	2,930	—
Verongida	***Aiolochroia crassa***	1	23,878	9,224	9.2
		2	13,788	4,651	—
		3	2,221	1,340	—
	***Aplysina fistularis***	1	30,546	11,801	9.5
		2	24,347	17,444	—
		3	20,318	14,235	—
		4	6,705	6,514	—
	***Aplysina insularis***	1	17,772	13,600	8.9
	***Aplysina lacunosa***	1	11,491	4,346	8.5
		2	9,080	3,689	—
	***Verongula gigantea***	1	76,641	9,792	10.4
		2	55,630	15,020	—
		3	53,736	18,884	—
		4	22,366	11,478	—
		5*	35,422	18,136	—
		5*	35,276	18,137	—
		5*	34,744	18,036	—
		5*	33,901	17,767	—
	***Verongula rigida***	1	8,855	19,262	8.2
		2	2,302	4,145	—

Among the 41 models, the initial camera alignment step produced sparse point clouds that had a minimum of 28,725 and average ($\pm$ SD) of 80,461 $\pm$ 35,891 tie points. Further processing produced dense point clouds that had a minimum of 2,403,071 and average of 10,163,668 $\pm$ 6,127,860 points. Average scale error was 0.48 $\pm$ 0.58 mm, and scale error did not exceed 2.63 mm (Table 2). The total time required to process all models (from image alignment to textured mesh) was 9,264 min (6.4 d), and the average time required to process each model was 234 $\pm$ 156 min (3.9 $\pm$ 2.6 h; macOS, 2.9 GHz Intel Core i7 processor, 16 GB RAM; Supplementary Fig. 1). Among four replicate scans of one individual of *V. gigantea*, averages of surface area and volume were 18,019 $\pm$ 174 cm${}^{2}$ and 34,840 $\pm$ 690 ml, respectively. The coefficients of variation (CV) for surface area and volume were 0.97% and 1.97% respectively.

**Table 2:** Summary of 3D reconstruction parameters, including definitions, averages, standard deviations (SD), minimums, and maximum values

Term	Definition	Mean	SD	Min	Max
# Images	Number of images in batch for an individual.	213	54	111	378
# Aligned Images	Number of images from batch that were able to be aligned	202	61	54	374
# Tie points	Number of points in the sparse cloud.	80,461	35,891	28,725	193,455
# Dense Cloud points	Number of points in dense cloud.	10,163,668	6,127,860	2,403,071	26,049,035
Flying Alt (cm)	Height above ground level.	59.6	15.1	37	116
Resolution (mm pix -1)	Effective ground resolution averaged over all aligned images.	0.24	0.07	0.15	0.49
Scale Error (mm)	Difference between input and estimated values for scale bar length	0.48	0.58	0	2.63
Reprojection Error (pix)	The distance between the point on the image where a reconstructed 3D point can be projected and the original projection of that 3D point detected on the photo.	0.85	0.08	0.58	0.99
Key Point Size	Mean tie point scale averaged across all projections.	4.14	0.24	3.5	4.45
Overlap	Mean number of projections of each point in the sparse point cloud.	3.51	0.66	2.5	4.97

When the exponential growth equation for *Aiolochroia crassa*^27^ was applied to the largest individuals from each of the 16 species, estimated ages ranged from 5.2 to 10.4 y (Table 1). The sponge species for which the estimated age most closely corresponded to the approximate age (based on the history of the wreck, see methods below) of 8.74 y were* Smenospongia aurea* (8.8 y), *S. conulosa* (8.6 y), *Aplysina insularis* (8.9 y), *Aplysina lacunosa* (8.5 y), and *Ailiochroia crassa* (9.2 y; Table 1). Among all individuals from 16 species, the average ($\pm$ SD) difference between estimated and approximate ages was 358 $\pm$ 332 d.

**Figure 3:**
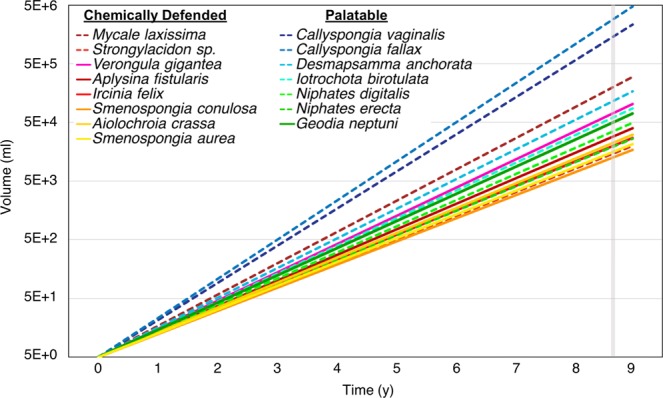
Growth forecast for each species. The volume measurements on the Y axis are plotted on a $lo{g}_{10}$ scale, starting at 5.0 ml (${V}_{0}$). The vertical gray line denotes 8.74 y. Solid lines represent specific growth rates of species surveyed on the Tibbetts wreck, and dashed lines represent specific growth rates of species surveyed on the Spiegel Grove wreck^4^.

A specific growth rate was calculated for seven of 16 species on the Tibbetts wreck (for which n > 2 individuals per species) and all eight species previously surveyed on the Spiegel Grove^4^. Of the seven species from the Tibbetts, the largest (*Verongula gigantea*) had a specific growth rate of 1.10 y${}^{-1}$, and the smallest (*Smenospongia conulosa*) had a specific growth rate of 0.90 y${}^{-1}$. The largest species on the Spiegel Grove (*Callyspongia fallax*) had a specific growth rate of 1.53 y${}^{-1}$, while the smallest (*Strongylacidon sp*.) had a specific growth rate of 0.92 y${}^{-1}$ (SI Table 1). A nine-year growth forecast indicated overlap among the species from each shipwreck; the predicted volumes for four of eight Spiegel Grove species (V${}_{8.74}$ = 15.6–65.1 L) were within the range of observed volumes of Tibbetts sponges (V${}_{8.74}$ = 13.4–76.6 L). The predicted volumes for the remaining four species from the Spiegel Grove (V${}_{8.74}$ = 125.4–3,181 L) exceeded the range of Tibbetts sponge volumes, and the two species from the Spiegel Grove whose predicted volumes deviated to the greatest extent were *C. fallax* (V${}_{8.74}$ = 3,181 L) and *C. vaginalis* (V${}_{8.74}$ = 2,317 L; Fig. 3). Six of the seven slowest growing species (SGR < 1 y${}^{-1}$) from both shipwrecks were previously determined to have tissue that was chemically defended against predatory fishes^28^: *Smenospongia conulosa* (Tibbetts), *Strongylacidon sp*. (Spiegel Grove),* Smenospongia aurea* (Tibbetts), *Ircinia felix* (Tibbetts), *Aiolochroia crassa* (Tibbetts), and *Aplysina fistularis* (Tibbetts). Conversely, six of the eight fastest growing species (SGR > 1 y${}^{-1}$) were previously determined to be palatable to predatory fishes^28^: *Callyspongia fallax* (Spiegel Grove), *Callyspongia vaginalis* (Spiegel Grove), *Desmapsamma anchorata* (Spiegel Grove), *Iotrochota birotulata* (Spiegel Grove), *Geodia neptuni*(Tibbetts), and *Niphates digitalis* (Spiegel Grove; Supplementary Table 1).

## Discussion

Basic demographic information about many benthic marine organisms is lacking because these organisms do not generate indicators of annual growth, such as the density bands found in the mineralized skeletons of some corals or the otoliths of many fishes. This study provides data on the volume (a proxy for biomass) at an approximate age (8.74 y) for 16 common species of sponges found on Caribbean reefs, as well as specific growth rates for seven of the most abundant of these species.

**Figure 4:**
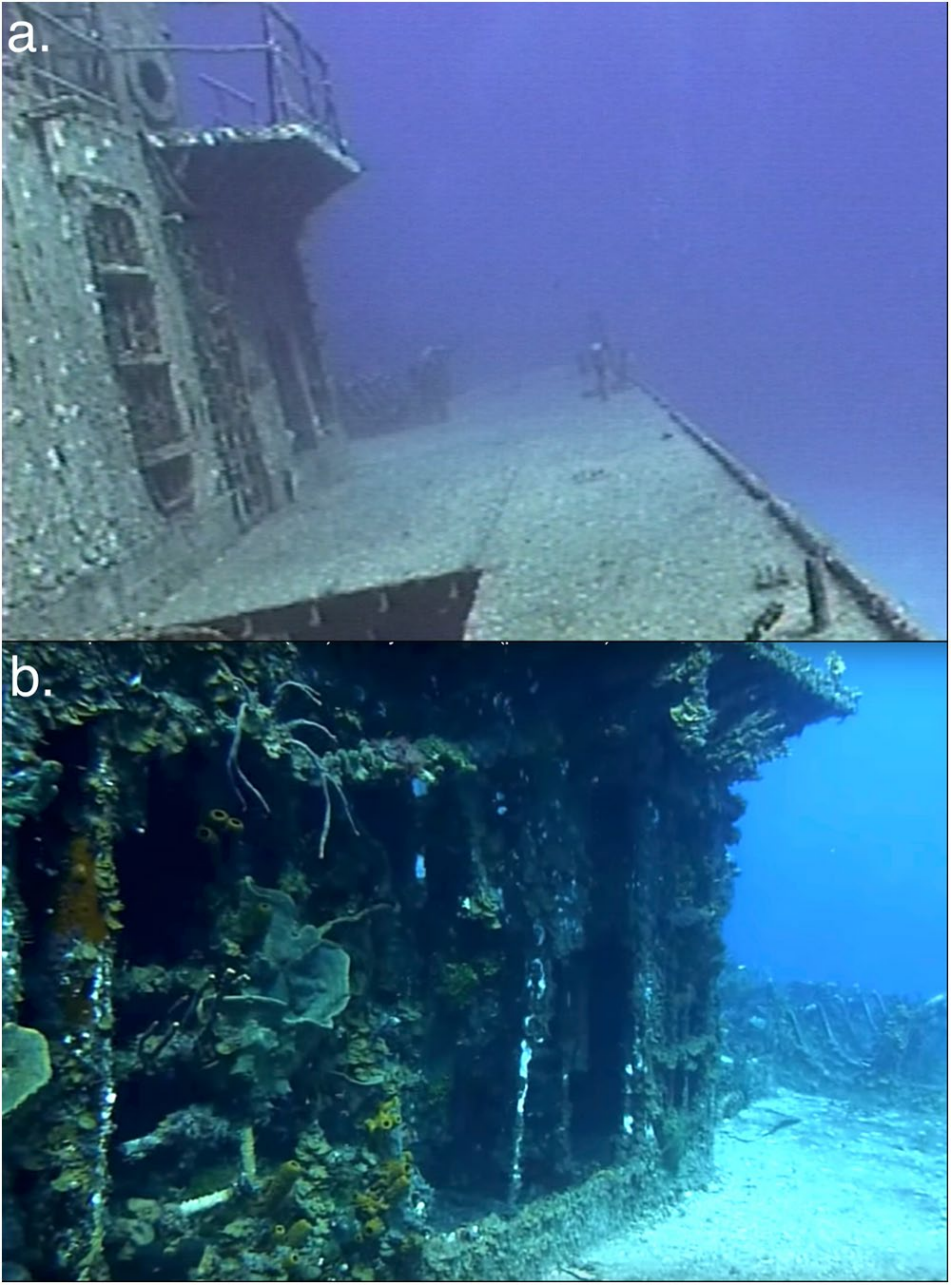
The progression of sponge community development pre- and post- hurricane Paloma, as seen from YouTube video excerpts showing the same door on the starboard side of the aft deck house, taken in (**a**) 2006 by S. Kitchen-McKinley and (**b**) 2010 by M. Sandison. Links to corresponding YouTube videos can be found in Table 3 (ref. ^2,3^).

We are reasonably confident in the validity of the assumptions made regarding the approximate age of the largest sponges from the Tibbetts wreck that have resulted in the specific growth rates reported herein. First, as explained in the methods, we assume that no sponges were present prior to 9 November 2008. Before this date, sponge community development was inhibited by the destructive effects of hurricanes, as evidenced by photographs of the wreck surface from video recordings (Table 3, Fig. 4). Of course, we cannot be certain that the sponges began growing from propagules that recruited prior to 9 November 2008, were ripped from the wreck during hurricanes, and subsequently grew from remaining small bits of tissue. If this were the case, however, it would have little effect on estimates of growth rate. Second, we assumed that all sponges originated from a single propagule, as opposed to recruiting as asexually produced fragments. These massive sponge species are not known to reproduce by fragmentation, as are some branching species. Even if fragmentation was possible, transport of fragments from reef to wreck was unlikely because the individuals that we surveyed grew on elevated and sloped surfaces. Third, we assumed a low likelihood of tissue loss due to storms or predation. Storm damage was responsible for delaying the onset of sponge community development through 9 November 2008, but there were no major storms after this date, and sponge community development was evident in videos taken in 2010, 2013, and 2016 (Table 3, Fig. 4). Loss of tissue due to predation was similarly unlikely to have occurred, because all but two species were known to be chemically defended from vertebrate predators^28^, and the two undefended species, *Geodia neptuni* and *Chondrosia reniformis*, have a tough outer cortex. While both of these undefended species are subject to predation by hawksbill turtles^10^, none of the specimens measured for this study showed evidence of turtle bites. Despite our confidence in the validity of the assumptions implicit in this study, the limitations of this approach to understanding sponge demographics should be recognized.

**Table 3:** YouTube videos used to construct a timeline of sponge community development on the Tibbetts shipwreck.

#	Year	URL	Title (Creator)	Screenshot
1	1998	youtu.be/8p0MXXz5G50	“Episode 46: Capt. Keith Tibbetts Wreck, Cayman Brac” (Don Stark)	2:24 (turret)
2	2006	youtu.be/oLKK0IAuw0Y	“Russian Frigate 356 Shipwreck Dive” (Kitchen1066)	4:31 (turret)
				6:32 (tower)
				5:03 (deck house)
3	2010	youtu.be/qOc7yj2GqTI	“M/V Captain Keith Tibbetts (356) in Cayman Brac (part 1 of 2)” (morays)	7:39 (deck house)
4	2010	youtu.be/rOEnZ6QHjE	“Diving the Tibbetts” (photobyace)	5:09 (tower)
5	2013	youtu.be/mUGvwYKbGw4	“Captain Keith Tibbetts Wreck (Russian Destroyer #356)” (Vance Esler)	3:45 (turret)
6	2016	youtu.be/aTuU8VnIseI	“Scuba diving Captain Keith Tibbetts wreck Cayman Brac” (JAYTHEDIVER)	1:05 (tower)
				2:42 (turret)

This study highlights the technological advantage of 3D photogrammetry for the determination of surface area and volume of benthic organisms. Negligible scale error (< 4%; Table 2) and minimal variation (CV$${}_{volume}$$ < 2% and CV$${}_{surface}$$$${}_{area}$$ < 1%) across the four replicate scans of *Verongula gigantea* demonstrated the accuracy and precision of these measurements, and the latter is comparable to similar precision metrics recently reported for coral skeletons (CV$${}_{volume}$$ = 7%, CV$${}_{surface}$$$${}_{area}$$ = 1.5%)^14^. The benefits conferred by photogrammetry over other techniques did not require a proportional increase in time or manpower. Imaging of sponges in the field was completed in one day, and while post-processing required seven days, this was largely automated. Photogrammetry is also non-destructive, a key improvement over traditional methods such as water displacement^3,4^ and wrapping with paraffin wax or tinfoil^5,6^. As an added benefit, 3D models are easily shared (https://sketchfab.com/l.olinger/collections/the-sponges-of-tibbetts-wreck-cayman-brac) for collaboration and public outreach. Despite the foregoing advantages, 3D models could not be made of all the sponge species found on the Tibbetts wreck. The purple rope sponge *Aplysina cauliformis*, one of the most abundant species in the Caribbean^28^, has a branching morphology, and the movement of branches of *A. cauliformis* in water currents led to failed image alignment for all three individuals that were photographed. Photogrammetric imaging of organisms that move will require still-water conditions or enhanced techniques, perhaps including synchronized imaging with a multi-camera system. Additionally, photogrammetric reconstructions are limited to the portions of the target organism that can be photographed. This limitation arose for models of *A. fistularis*, whose atria could not be resolved with the photogrammetric procedure and thus had to be accounted for after the fact.

To date, only a few studies have attempted to estimate sponge age or model growth^8,25– 27^. In this study, we applied one existing model describing the growth of *Aiolochroia crassa*^27^ to the 16 sponge species on the Tibbetts wreck. Encouragingly, the predicted age for *Aiolochroia crassa* from the Tibbetts wreck deviated very little (172 d) from the assumed age of 8.74 y. Additionally, the model was fairly accurate at predicting the ages of other sponge species belonging to the orders Verongida and Dictyoceratida (Table 1). Similarities in the growth rates among these species are likely due to their shared characteristics: all are high microbial abundance, or HMA, sponges^29^ with non-mineralized skeletons^30^ and chemical defenses^28^. It should be noted that, because of the steep shape of the curve of the *Aiolochroia crassa* growth model, any volumes between approximately 5 and 40 L will correspond to an age that is within one year of the assumed age of 8.74 y (SI Fig. 2). Nevertheless, we observed an even narrower range of volumes (<10 L; V = 11.5 – 21 L) for the five species whose predicted age deviated the least (<120 d) from the approximate age of 8.74 y (Table 1).

Other attempts have been made to model sponge growth using size-at-age formulae. In one, a generalized von Bertallanfy growth function (gVBGF) was constructed from populations of the giant barrel sponge, *Xestospongia muta* from the Florida Keys^8^. Unlike the sponge species measured on the Tibbetts wreck, *X. muta* is subject to grazing from parrotfishes^31^, and growth may be highly variable over time, with some individuals observed to shrink in size^8^. Additionally, data used to derive the gVBGF for *X. muta* included individuals spanning a much larger range of sizes and ages^8^ relative to the sponges measured herein. Not surprisingly, therefore, application of the gVBGF function to volume data for the largest sponges from the Tibbetts wreck returned age estimates of 35 years for *Verongula gigantea* and 21 years for *Aiolochroia crassa* (Appendix S1; Supplementary Table 1); the recruitment years derived from these estimates impossibly predate even the sinking of the wreck. A recent attempt to model the growth of Indo-Pacific *Xestospongia spp*. reported specific growth rates similar to those for Florida *X. muta* (0.47 vs 0.52, respectively) but a steeper growth curve indicative of much faster growth^26^. It is not clear why such discrepancies should occur given similar proportional growth rates; one explanation may be that the shorter experimental duration (2 y vs. 4.5 y, respectively) used in the Indo-Pacific study resulted in growth estimates that were obscured by other sources of error (including variable growth rates mentioned above) and subsequently poorer model fit (as evidenced by large standard errors). It also cannot be ruled out that both models are correct and growth rates vary between Indo-Pacific *Xestospongia spp*. and Florida *X. muta* due to inherent species- and site-level differences. Similar species-level differences likely introduced error in the age estimates derived from the *A. crassa* growth model that were reported herein. Additionally, because proportional growth rates decrease as sponges age^32^, an exponential growth equation may be a poorer fit for older sponges. The foregoing limitations are why predictions from the *A. crassa* growth models are accompanied by secondary observations (i.e., videos of the shipwreck) supporting an estimated age of 8.74 y. Although informative, the validity of growth models are largely restricted to the species they were built for, and limited by the quality of data used to parameterize them.

This is the second study to describe Caribbean sponge community development on a shipwreck, and there were important differences between the present study of the Tibbetts wreck and that of the Spiegel Grove^4^ besides the techniques used for volume determination (photogrammetry vs. water displacement, respectively). The Spiegel Grove wreck was quickly colonized by fast-growing, palatable sponge species when it was surveyed, approximately 4.4 years after sponge community development began^4^. The Tibbetts wreck was surveyed approximately 8.7 years after sponge community development, and while some palatable sponge species were present on the wreck (e.g., *Iotrochota birotulata*), these clearly exhibited grazing scars. The difference in sponge community between the two studies was likely due to differences in the location of each wreck and more than two-fold difference in the age of the sponge community. The Spiegel Grove wreck was upright, approximately 800 m from the nearest reef, and with the horizontal decks 15–23 m above the sandy bottom. As such, the wreck had not attracted common reef fishes including sponge-eating angel- and parrotfishes at the time of the survey; indeed, it was noted for attracting sharks and large groupers, which are predators of sponge-eating fishes. The Tibbetts wreck, on the other hand, was sunk just tens of meters from adjacent coral reef, with surfaces only a few meters above the sandy bottom, allowing sponge-eating fishes easy access. Therefore, in the absence of sponge predators, the Spiegel Grove wreck was dominated by fast-growing, palatable sponge species, but the Tibbetts was not. Notably, when the Spiegel Grove wreck was visited 18 months after initial surveys, the sponge community had attracted sponge-eating angelfishes and was transitioning to more chemically defended species^4^, like those found on the Tibbetts wreck.

There is ample experimental and correlative evidence to support a resource trade-off between tissue growth and chemical defenses in Caribbean sponges^33,34^, and comparative analyses of the specific growth rates of sponges from both the Tibbetts and Spiegel Grove wrecks provide further support for this hypothesis. Specific growth rates were generally much faster for palatable sponge species than for chemically defended species. Palatable sponges such as *Callyspongia vaginalis*and *Callyspongia fallax* grew remarkably fast relative to other sponge species, with specific growth rates of 1.45 and 1.53, respectively (SI Table 1). Growth forecasts based on the specific growth rates predicted that *C. vaginalis* and *C. fallax* would attain volumes of over 2000 and 3000 L, respectively, after nine years of growth (Fig. 3). These species generally do not reach such large sizes because of the compensatory effects of grazing by sponge-eating fishes or because of tissue loss from storm events. However, particularly large specimens of *Callyspongia vaginalis* can be found on the reefs of Bocas del Toro, Panama, a location that is both heavily overfished and infrequently subjected to storms^2^.

While the growth rate of most species was correlated to whether they produced chemical defenses, there were some exceptions. For example, growth of the undefended species *Niphates erecta* was uncharacteristically slow, and this has been reported in previous studies^33^. Additionally, growth of the chemically defended species *Mycale laxissima* was rapid and more closely resembled that of palatable species. *Mycale laxissima* was also the only species found on both the Tibbetts and Spiegel Grove wrecks, but comparisons between shipwrecks could not be made because only two small individuals of *M. laxissima* were identified on the Tibbetts (Table 1). This species is known from previous studies to have rapid growth, frequent reproduction and high mortality rates^32^, so it is possible that the two small individuals of *M. laxissima* from the Tibbetts recruited much later than November 2008.

The fortuitous circumstances of being able to apply 3D photogrammetry to a well-documented shipwreck enabled us to measure the volume and surface area and to approximate the age of 16 species of common Caribbean reef sponges. These measurements and age approximations were validated through comparison with ages predicted using an exponential growth model for *Aiolochroia crassa*^27^, which showed a surprising degree of accuracy. Furthermore, specific growth rates derived from volume measurements and approximate age, when compared to specific growth rates for sponges from the Spiegel Grove wreck^4^, added to the considerable body of evidence for a resource trade-off between sponge growth rate and the production of chemical defenses that deter vertebrate predators. The present study demonstrates that the demographics of modular and clonal benthic marine organisms, such as sponges, can be better understood through the investigation of their community development on anthropogenic structures combined with the use of rapidly developing technologies, such as crowd-sourced video and 3D imaging using photogrammetry.

## Methods

### Imaging of sponges on the Tibbetts shipwreck

The MV Captain Keith Tibbetts shipwreck is a Soviet-era frigate, 101 m in length, that was scuttled off the island of Cayman Brac, in the Cayman Islands, in September 1996. The wreck is located 183 m from shore, and the bow and stern are situated at depths of 26 m and 18 m, respectively. Videos of the largest (by volume) individual sponges growing on the Tibbetts were collected on SCUBA between August 6-7, 2017. Before taking video, a 30.5 cm ruler was affixed to the sponge to be filmed. Short (1-2 min) videos were then collected using a GoPro camera (GoPro Hero 5, 4K video resolution, Wide FOV, 30 fps) that was held ~60 cm distant and rotated to capture all possible perspectives of the sponge and ruler. Later, single frames were extracted from every 15–20th frame of the video and together these represented an image set. Image sets were inspected, and redundant, blurry, poorly lit, or distant photographs were removed. The white balance, tone, temperature, and vibrancy of image sets were batch edited.

### Photogrammetry processing

Digital models were produced using the photogrammetric software Agisoft PhotoScan Professional edition (Agisoft L.L.C., 2013). Image sets were imported into the software and then aligned (high accuracy, key point limit 40,000, tie point limit 10,000, pair preselection enabled). Alignment is an automated process in which the software detects points that are visible in at least two photographs (i.e., tie points)^35^ and projects these in 3D space as a sparse point cloud. The accuracy setting determined whether images were scaled prior to alignment, with “high accuracy” keeping images at their original size. The remaining three parameters were defined on the basis of minimizing processing time. The key and tie point limits represented the maximum number of points to be sampled from each image and the maximum number of points needed to match two images together, respectively, while enabling pair preselection allowed for the creation and alignment of image pairs.

Camera calibration (i.e., estimation of intrinsic camera parameters) was done automatically as part of the proprietary algorithm used in PhotoScan^36^. The process of auto-calibration began by setting the parametric lens distortion model to “fisheye” before image alignment, to accommodate distortion inherent to the wide-angle images captured by GoPros. For this type of calibration, it is also recommended to have strong camera geometry and ample image overlap, which we accomplished by extracting frames from high frame rate videos that showed all angles of the target object. Image alignment produced initial estimates for camera calibration parameters, and these estimates were refined using subsequent gradual selection and camera optimization steps, as recommended. First, points with a reconstruction uncertainty >25 were eliminated; this sorted points whose 3D locations were estimated with high uncertainty, for example when projected only from photographs taken at parallel angles. This also helped to remove points whose locations were projected from image peripheries, where fisheye distortion was the most severe. The optimize cameras tool was then used to refit the positions of the remaining “good” points and provide refined estimates for camera calibration parameters. Next, points with a reprojection error > 1 pixel were eliminated; this filtered points whose 3D locations deviated more than one pixel from the locations on the photographs they were projected from. Finally, points with a projection accuracy >10 were eliminated; this filtered points that were matched from photographs taken from drastically different distances from the target object.

The next step was generation of a dense point cloud (high quality, depth filtering disabled). This step was the most computationally demanding, but performance was improved with GPU acceleration (GPU devices: AMD Radeon Pro 455 Compute Engine with 12 compute units @ 855 MHz 2048 MB and IntelRHD Graphics 530 with 24 compute units @1050 MHz 1536 MB). Upon completion, the dense clouds were visually inspected, and the manual selection tool was used to remove extraneous points. A mesh (average one million faces, surface type arbitrary, face count high) was then made through interpolation of the remaining points in the dense cloud, and finally the photographic detail was projected onto the newly generated surface by creating a texture (mapping mode generic, blending mode average).

The final step in PhotoScan was to scale the model by making two scale bars (length = 8–10 cm) on either end of the 30.5 cm ruler. This began by searching the individual images for one with a sharply defined view of the ruler. Once found, the scale bars were then demarcated on the image, first by placing markers (the end points of the scale bars) on the tips of the millimeter ticks of the ruler, and then by defining the lengths between the markers using the scale bar function. The mesh and other images were then inspected to verify each markers’ position corresponded to that on the image on which it was marked, and scale error was reported from the PhotoScan reference pane.

### Photogrammetry post-processing and volume measurement

**Figure 5:**
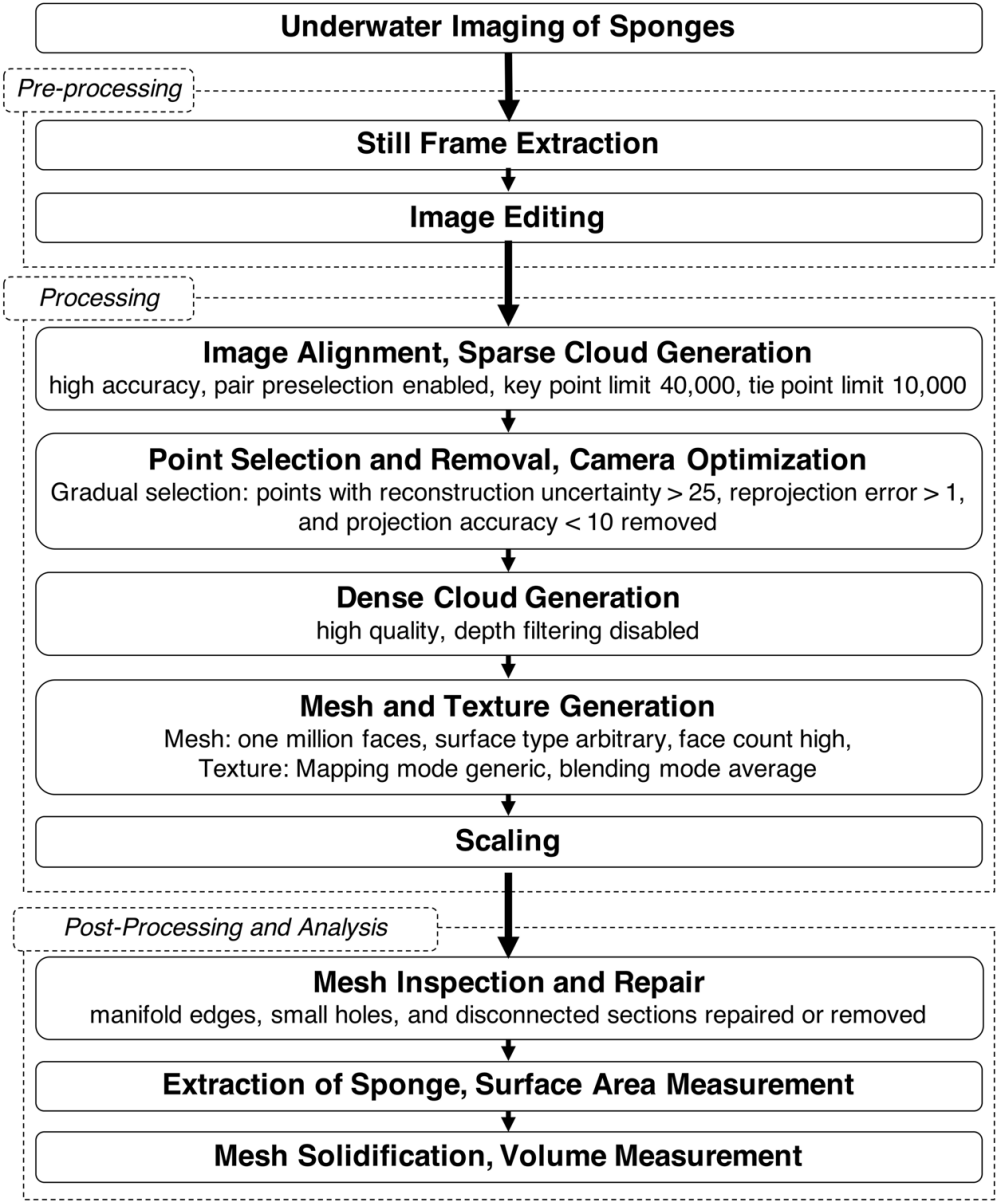
Photogrammetric Workflow

Each scaled and textured mesh was imported into Autodesk Meshmixer (Autodesk, Inc. 2017). First, the inspector tool was used to examine the mesh and repair or remove manifold edges, small holes, and extraneous mesh pieces that were artifacts of the reconstruction process. Manual selection was then used to extract the sponge surface from the rest of the 3D mesh, and the surface area of this extracted, open-faced mesh was measured. To solidify the mesh, the bridge or join tool was used to superimpose a surface at the base of the extracted sponge representing the boundary between sponge and substratum, and then the volume was measured. This complete workflow is summarized in Fig. 5.

In some cases, the sponge was growing around an object such as a guard-rail or bundle of cables. To prevent inclusion of these non-sponge objects in final volume measurements, a 3D object with the approximate dimensions of the intersecting rail or cables was generated, aligned to the intersecting object, and its volume extracted from the isolated sponge using a Boolean difference operation (e.g., Supplementary Fig. 3). The model was then re-inspected and volume measured. Because the reconstruction process did not accurately depict the negative space representing the atria of the tube sponge *Aplysina fistularis*, atria volumes were calculated using the equation

1 $${V}_{atria}={\sum }_{i=0}^{n}\pi {\left(\frac{D}{2}\right)}^{2}H$$

where $n$ = number of tubes, $D$ = maximum oscula diameter, and $H$ = tube height. The total atrial volume (${V}_{atria}$) was then subtracted from the volume of the solidified mesh.

### Precision analysis

To evaluate the precision of the volume and surface area measurements collected from 3D models, four videos were recorded of the same individual of *Verongula gigantea*. This species was chosen because of its abundance on the wreck and high surface area to volume ratio, which makes for a robust test of 3D precision. Four models were generated from the four videos following the same processing workflow used throughout the rest of this study (Fig. 5). The sponges were extracted from each of the models and aligned with one another to ensure that the extracted models had similar boundaries. Then the surface areas (of open meshes) and volumes (of solid meshes) were measured.

### Estimation of maximum age of sponges from YouTube videos

We approximated the maximum total age (from recruitment to time of imaging for photogrammetry) of the largest individuals of each sponge species by conducting a search of publicly-available YouTube videos of the shipwreck. From this, we assembled a timeline of video excerpts from 1998 to 2016 that showed the aft gun turret, conning tower, and deck house from similar camera angles. Similar crowd-sourcing of data from YouTube videos has been used recently to monitor the rise of the water level in a Saudi Arabian cave^37^. Our timeline reveals no sponges between 1998 and 2006, but the presence and growth of sponges is evident as early as 2010 and through 2016 (Table 3, Fig. 4). A delay of over a decade between the sinking of the wreck (in 1996) and the start of sponge community development was unexpected, compared to studies from other shipwrecks. For example, sponges were abundant on the decks of the Spiegel Grove only four years after the sinking of the wreck^4^. The delay, which can be seen in Fig. 4, was likely caused by a series of hurricanes that impacted Cayman Brac between 1998 and 2008: Lili (2002), Charley (2004), Ivan (2004), and Paloma (2008). The latter two storms –Ivan and Paloma – were notable for splitting the wreck in two and causing extensive damage to the island of Cayman Brac, respectively^38^. Combining photographic evidence and the storm history, we approximated the earliest date of sponge larval settlement onto the wreck as 9 November 2008 – the day after the most recent hurricane (Paloma) cleared the island of Cayman Brac. The duration between 9 November 2008 and 4 August 2017 (when sponges were photographed for this study) was 8.74 y; this is the approximate age of the largest and therefore theoretically oldest individuals on the Tibbetts wreck.

### Age estimation from an exponential growth model

An exponential equation describing the growth of *Aiolochroia crassa*^27^ was used to estimate the age of the largest individual of each species. We chose this equation because of the presence of *A. crassa* and other verongid sponge species on the wreck. The equation for the exponential growth equation is

2 $${W}_{t}=A{e}^{a}t$$

where ${W}_{t}$ is the weight at time $t$, $A$ is the growth constant, and $a$ is relative growth rate. For ${W}_{t}$, the volume of each individual was converted to wet weight assuming a volume to wet weight ratio of 0.94 mL to 1 g^27^. The remaining variables – growth constant $A$ and relative growth rate $a$ – were held constant at the original values describing the growth of *A. crassa* ($A$ = 2.567, $a$ = 0.0027)^27^. Equation (2) was solved for $t$ to calculate the age of each individual and compare it to the assumed age of 8.74 y.

### Specific growth rates

For the most prolific sponge species growing on the Tibbetts wreck, specific growth rates (SGR) of the largest individuals were determined exponentially, using the equation

3 $$SGR=\frac{\left(ln(\frac{{V}_{t}}{{V}_{0}})\right)}{{d}_{t}}$$

where ${V}_{t}$ is the volume (of the largest individual), ${V}_{0}$ is the initial volume (${V}_{0}$= 5.0 ml), and ${d}_{t}$ is the assumed age (${d}_{t}$ = 8.74 y). We only included in this analysis the species that were represented by the largest and most abundant individuals on the wreck (in other words, any species from those surveyed that were represented by > 2 individuals) because abundance suggests higher recruitment frequency and therefore greater likelihood that the largest individual settled nearer to 9 November 2008. Equation (3) was also used to calculate specific growth rates for all of the sponge species growing on the Spiegel Grove, a shipwreck located in Florida Keys, and whose volumes were previously measured by collection and water displacement^4^. Similar to specific growth rate calculations for the Tibbetts sponges, ${V}_{t}$ was set equal to the volume of the largest individual and ${V}_{0}$ was set to an initial volume of 5 ml. The earliest date of sponge larval settlement on the Spiegel Grove was approximated to be the date of sinking (11 June 2002), and sponges were collected and volumes measured between October 10 and November 14, 2006^4^. Subtracting the approximate settlement date from the dates that sponges were collected gave approximate ages (${d}_{t}$) ranging from 4.33 to 4.35 y. The specific growth for species from both shipwrecks were used to construct a nine-year growth forecast, and the predicted volumes of Spiegel Grove sponges at t = 8.74 y (V${}_{8.74}$) were compared to the measured volumes of the Tibbetts sponges. All sponge species identified on the Tibbetts and Spiegel Grove were categorized as having tissue that was either chemically defended against predatory fishes or palatable to predatory fishes (consistently undefended or variably defended)^28^.

## Supplementary information

Supplementary Information

## Data Availability

All data generated or analyzed during this study are included as supplementary information. Additionally, all 3D models are available at https://sketchfab.com/l.olinger/collections/the-sponges-of-tibbetts-wreck-cayman-brac.
