# Supplementary material for: Growth estimates of Caribbean reef sponges on a shipwreck using 3D photogrammetry
Source: Sci Rep. 2019 Dec 5;9:18398. doi: 10.1038/s41598-019-54681-2 (PMC6895235; doi:10.1038/s41598-019-54681-2)
Supplement: Supplementary file 1 — Supplementary Information [file 41598_2019_54681_MOESM1_ESM.docx]

**﻿**Growth estimates of Caribbean reef sponges on a shipwreck using 3D photogrammetry

Lauren K. Olinger, Alexander R. Scott, Steven E. McMurray, Joseph R. Pawlik

# Supplementary Information

**Supplementary Table 1** Approximated ages (d_t_), measured volumes (V), and specific growth rates (SGR) of the largest individual of each sponge species from the Tibbetts and Spiegel Grove shipwrecks. Chemically defended species in bold

| **Shipwreck** | **Order** | **Species** | **d_t_ (y)** | **V (ml)** | **SGR** |
| --- | --- | --- | --- | --- | --- |
| Tibbetts | Astrophorida | *Geodia neptuni* | 8.74 | 53,296 | 1.061 |
|  | Dictyoceratida | ***Ircinia felix*** | 8.74 | 21,047 | 0.955 |
|  |  | ***Smenospongia aurea*** | 8.74 | 16,663 | 0.928 |
|  |  | ***Smenospongia conulosa*** | 8.74 | 13,387 | 0.903 |
|  | Verongida | ***Aiolochroia crassa*** | 8.74 | 23,878 | 0.969 |
|  |  | ***Aplysina fistularis*** | 8.74 | 30,546 | 0.997 |
|  |  | ***Verongula gigantea*** | 8.74 | 76,641 | 1.103 |
| Spiegel Grove (Pawlik et al. 2008) | Haplosclerida | *Callyspongia fallax* | 4.44 | 3,840 | 1.529 |
|  |  | *Callyspongia vaginalis* | 4.43 | 2,720 | 1.450 |
|  |  | *Niphates digitalis* | 4.33 | 420 | 1.022 |
|  |  | *Niphates erecta* | 4.33 | 310 | 0.952 |
|  | Poecilosclerida | *Iotrochota birotulata* | 4.33 | 550 | 1.084 |
|  |  | ***Strongylacidon sp.*** | 4.33 | 270 | 0.920 |
|  |  | *Desmapsamma anchorata* | 4.33 | 760 | 1.159 |
|  |  | ***Mycale laxissima*** | 4.35 | 1,010 | 1.222 |

**Appendix S1 – Age estimations of Tibbetts sponges using the generalized von Bertalanffy growth function (gVBGF; McMurray et al. 2008)**

The ages of all individuals were estimated using a generalized von Bertalanffy growth formula (gVBGF) and previously published parameter estimates for *X. muta* (McMurray et al., 2008). The equation for the gVBGF is

$$S_{2}={(S_{\infty}^{1/D}\left( 1-e^{-K*dt} \right)+ S_{1}^{1/D}\left( e^{-K*dt} \right))}^{D}$$

where S_∞_ is the cube root of the volume reached at infinite period of growth, *K* is the relative growth rate, and *D* determines the shape of the curve. Equation 4 was solved for *dt* in order to calculate the age of each individual. The cube root of the volume of each individual was assigned to *S*_2_, and the remaining variables were assigned their original values reflecting the growth of *X. muta* (S_∞_ = 66.1211, *K* = 0.0388, *D* = 1.8972, S_1_ = 2.89) (McMurray et al., 2008).

**Supplementary Table 2** Volumes and surface areas for each individual, and estimated ages for the largest individuals from each species from the exponential growth model (Wilkinson and Cheshire 1988) and gVBGF (McMurray et al. 2008). Chemically defended species in bold

| **Order** | **Species** | **#** | **V (mL)** | **SA (cm2)** | **Estimated age (exponential growth model)** | **Estimated age (gVBGF)** |
| --- | --- | --- | --- | --- | --- | --- |
| Agelasida | ***Agelas dilitata*** | *1* | 5,954 | 3,647 | 7.8 | 12.53 |
|  |  | *2* | 4,984 | 3,298 | - | - |
| Astrophorida | *Geodia neptuni* | *1* | 53,296 | 10,470 | 10.0 | 29.38 |
|  |  | *2* | 15,863 | 3,990 | - | - |
|  |  | *3* | 14,257 | 3,115 | - | - |
| Chondrosida | *Chondrosia reniformis* | *1* | 5,919 | 2,357 | 7.8 | 12.50 |
| Dictyoceratida | ***Ircinia felix*** | *1* | 21,047 | 4,918 | 9.1 | 20.07 |
|  |  | *2* | 18,525 | 5,214 | - | - |
|  |  | *3* | 7,436 | 2,197 | - | - |
|  | ***Smenospongia aurea*** | *1* | 16,663 | 6,667 | 8.8 | 18.36 |
|  |  | *2* | 13,001 | 7,774 | - | - |
|  |  | *3* | 2,516 | 2,508 | - | - |
|  | ***Smenospongia conulosa*** | *1* | 13,387 | 5,086 | 8.6 | 16.91 |
|  |  | *2* | 9,729 | 3,000 | - | - |
|  |  | *3* | 8,436 | 3,553 | - | - |
| Halichondrida | ***Ptilocaulis walpersi*** | *1* | 479 | 1,270 | 5.2 | 4.50 |
|  |  | *2* | 196 | 383 | - | - |
| Homosclerophorida | ***Plakortis sp.*** | *1* | 6,569 | 2,391 | 7.9 | 12.99 |
| Poecilosclerida | ***Neofibularia nolitangere*** | *1* | 3,245 | 1,732 | 7.2 | 13.57 |
|  | ***Mycale laxissima*** | *1* | 1,645 | 1,364 | 8.0 | 9.99 |
|  |  | *2* | 7,390 | 2,930 | - | - |
| Verongida | ***Aiolochroia crassa*** | *1* | 23,878 | 9,224 | 9.2 | 21.08 |
|  |  | *2* | 13,788 | 4,651 | - | - |
|  |  | *3* | 2,221 | 1,340 | - | - |
|  | ***Aplysina fistularis*** | *1* | 30,546 | 11,801 | 9.5 | 23.24 |
|  |  | *2* | 24,347 | 17,444 | - | - |
|  |  | *3* | 20,318 | 14,235 | - | - |
|  |  | *4* | 6,705 | 6,514 | - | - |
|  | ***Aplysina insularis*** | *1* | 17,772 | 13,600 | 8.9 | 18.81 |
|  | ***Aplysina lacunosa*** | *1* | 11,491 | 4,346 | 8.5 | 15.97 |
|  |  | *2* | 9,080 | 3,689 | - | - |
|  | ***Verongula gigantea*** | *1* | 76,641 | 9,792 | 10.4 | 34.83 |
|  |  | *2* | 55,630 | 15,020 | - | - |
|  |  | *3* | 53,736 | 18,884 | - | - |
|  |  | *4* | 22,366 | 11,478 | - | - |
|  |  | *5** | 35,422 | 18,136 | - | - |
|  |  | *5** | 35,276 | 18,137 | - | - |
|  |  | *5** | 34,744 | 18,036 | - | - |
|  |  | *5** | 33,901 | 17,767 | - | - |
|  | ***Verongula rigida*** | *1* | 8,855 | 19,262 | 8.2 | 14.50 |
|  |  | *2* | 2,302 | 4,145 | - | - |


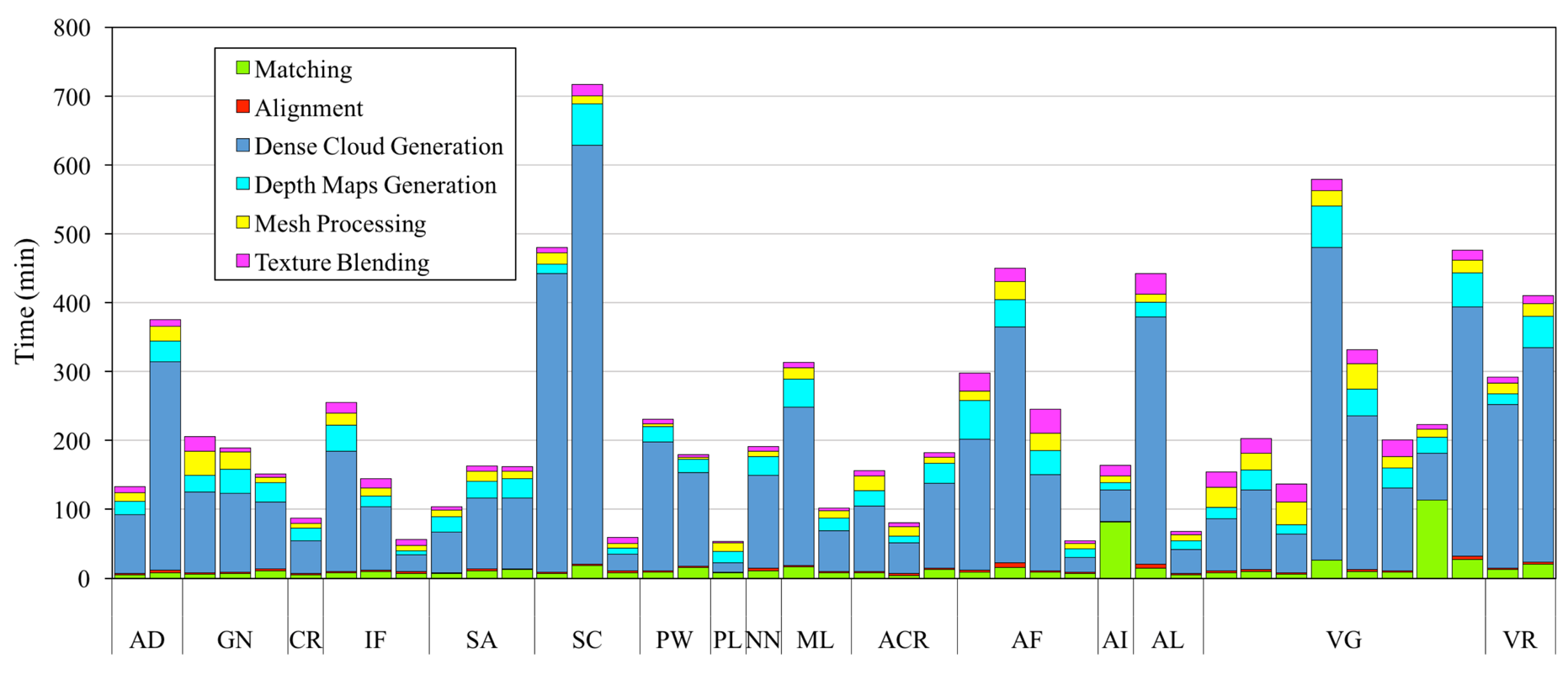


**Supplementary Figure 1** Time required to process each mesh, separated by reconstruction step. Individuals sorted into species. AD = *Ag. dilitata*, GN = *G. neptuni*, CR = *C. reniformis*, IF = *I. felix*, SA = *S. aurea*, SC = *S. conulosa*, PW = *P. walpersi*, PL = *Plakortis sp.*, NN = *N. nolitangere*, ML = *M. laxissima*, ACR = *Ai. crassa*, AF = *Ap. fistularis,* AI = *Ap. insularis*, AL = *Ap. lacunosa*, VG = *V. gigantea*, VR = *V. rigida.*


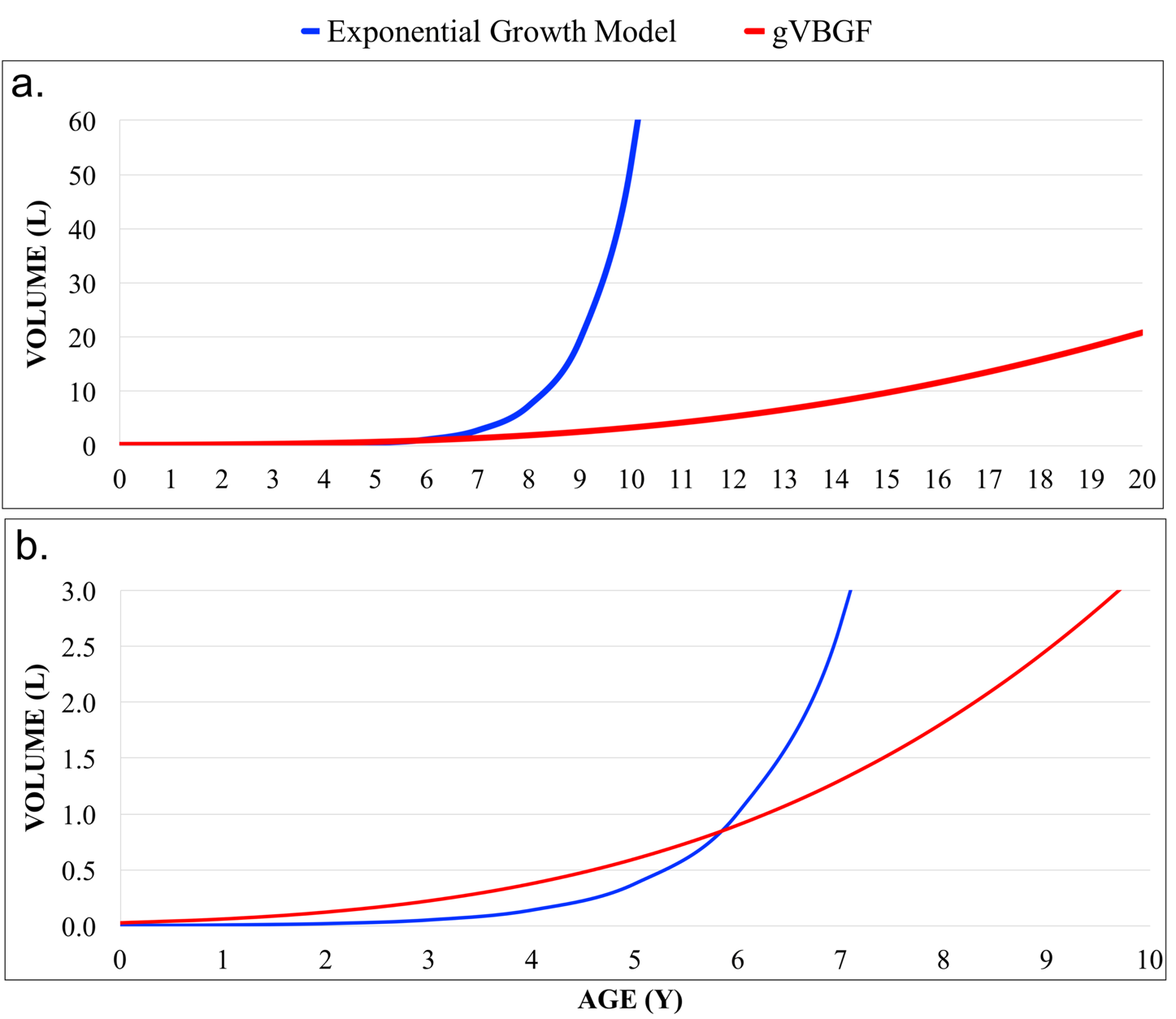


**Supplementary Figure 2** Size at age estimates from the exponential growth model for *A. crassa* (Wilkinson and Cheshire 1988), and gVBGF with parameters for *X. muta* (McMurray et al. 2008). a) estimates for sponges up to 20 yo; b) estimates for sponges up to 10 yo.


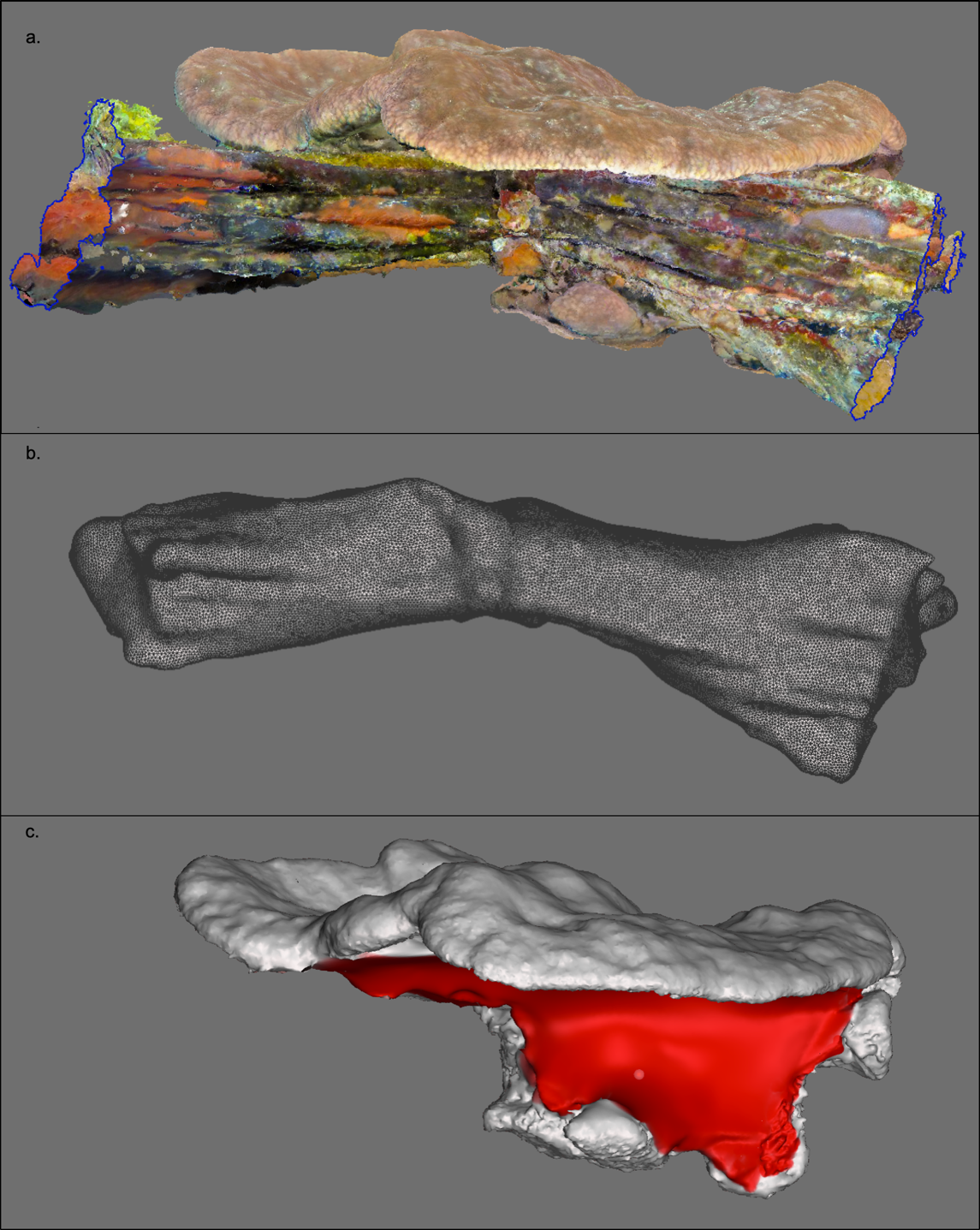


**Supplementary Figure 3** 3D model of *Agelas dilitata* a. Textured mesh showing sponge wrapped around bundle of cables; b. 3D object representing bundle of cables; c. Water-tight mesh showing isolated *A. dilitata* after Boolean difference operation to subtract volume representing cables. Surface created by Boolean operation highlighted in red. The textured mesh can be viewed at <https://skfb.ly/6wRRP>

**References**

McMurray, S. E., Blum, J. E. & Pawlik, J. R. Redwood of the reef: growth and age of the giant barrel sponge *Xestospongia muta* in the Florida Keys. *Mar. Biol.* 155, 159–171, https://doi.org/10.1007/s00227-008-1014-z (2008).

Pawlik, J. *et al.* Patterns of sponge recruitment and growth on a shipwreck corroborate chemical defense resource trade-off.  *Mar. Ecol. Prog. Ser.* 368, 137–143, https://doi.org/10.3354/meps07615 (2008).

Wilkinson, C. & Cheshire, A. Growth rate of Jamaican coral reef sponges after Hurricane Allen. *The Biol. Bull.* 175, 175–179, https://doi.org/10.2307/1541905 (1988).
